# The Risk Factors of Self-Management Behavior among Chinese Stroke Patients

**DOI:** 10.1155/2023/4308517

**Published:** 2023-03-11

**Authors:** Huang Shuqi, Li Siqin, Wu Xiaoyan, Yang Rong, Zhao Lihong

**Affiliations:** ^1^West China School of Nursing, Sichuan University, Chengdu 610041, China; ^2^Department of Liver Surgery, West China Hospital, Sichuan University, Chengdu 610041, China; ^3^Department of Pulmonary and Critical Care Medicine, West China Hospital, Sichuan University, Chengdu 610041, China; ^4^Department of Radiology, West China Hospital, Sichuan University, Chengdu 610041, China

## Abstract

**Background:**

Stroke is associated with a high number of disability-adjusted life years globally, so long-term care is necessary and important for those survivors, so self-management is becoming a more significant concept in stroke rehabilitation.

**Methods:**

Ischemic stroke patients (*n* = 354) were enrolled from the outpatient department of Neurology in West China Hospital from September 2018 to December 2019. The general demographic and disease-related data of stroke patients were collected. The stroke self-efficacy questionnaire (SSEQ), the brief cognition questionnaire (BIPQ), and the stroke self-management scale (SSMS) were used to collect data on self-efficacy, disease cognition, and self-management behavior separately. The chi-square test, Fisher exact test, independent sample *t*-test, and Mann–Whitney *U* test were used for comparison among groups. The logistic regression analysis was used to explore the independent risk factors of the different levels of self-management behavior in stroke patients.

**Results:**

The score of self-management among Chinese stroke patients was 151.07 ± 18.53. Multivariate analysis showed that the way of paying medical expenses (OR = 3.215, 95% CI (1.130, 7.769)), self-management efficacy (OR = 2.467, 95% CI (1.534, 3.968)), health education before discharge (OR = 2.354, 95% CI (1.457, 3.802)), age (elder) (OR = 2.060, 95% CI (1.265, 3.355)), educational level (OR = 1.869, 95% CI (1.169, 2.988)), and mRS score (OR = 1.850, 95% CI (1.129, 3.031)) were statistically significant (*P*  <  0.05).

**Conclusions:**

The self-management behavior of Chinese stroke patients was at the middle level. Patients with medical insurance, high self-efficiency of management, and better limb function may have better self-management behavior. Besides, patients with a high educational level who accept health education before discharge may also have better self-management behavior. For patients, it is important to know this disease in the right way and set up the faith to take care of themselves independently gradually. For medical staff, it is necessary and important to give all patients health education about self-management before discharge. It is urgent to call for attention to this disease, and the government and all of society should give more support to stroke patients.

## 1. Introduction

As one of the most important cerebrovascular diseases, stroke has been the second-most fatal and third-most disabling disease in the world, but it has been the leading cause of death in China in recent years, where almost one-fifth of the world's population resides [1–3]. A newly performed comprehensive assessment of the trends in China from 2013 to 2019 found the prevalence of stroke in China and most provinces has continued to increase in the past 7 years (2013–2019) and warrants a broad-based nationwide strategy for improved prevention as well as greater efforts in screening and more effective and affordable interventions [4]. Although in-hospital outcomes have improved because of a greater availability of evidence-based therapies and supportive care, adherence to secondary prevention strategies and long-term care are inadequate [5]. According to the scientific statement for health care professionals from the American Heart Association, there is strong evidence that self‐management is effective in achieving the goals of the treatment plan and cannot be ignored [6]. Furthermore, behavior, especially self-management or self-care behavior, is thought to be the predominant factor affecting illness and disease. Understanding self-management, knowing the risk factors of stroke, having a healthy diet consisting of physical activity and exercise, adhering to medication, and correcting unhealthy lifestyles are part of the self-management behavior of stroke patients. However, in China, medication adherence is poor among community-dwelling patients [7], and the necessary monitoring of stroke patients after discharge is scarcely in practice; besides, about 30 to 60% of patients do not have access to rehabilitation in hospitals [5]. Despite all this, it is hard to know the whole level of self-management behavior from single aspects of self-management behavior among Chinese stroke patients. Therefore, the purpose of this study is to describe the actual situation of Chinese stroke patients as a whole and explore the risk factors of self-management behavior.

## 2. Materials and Methods

### 2.1. Study Design and Participants

Stroke patients were continuously enrolled in West China Hospital, Sichuan University, from September 2018 to December 2019 and evaluated face-to-face at a clinic of the neurology department in this prospective cross-sectional study. The study was approved by the Ethics Committee of West China Hospital (2018496) and registered before conducting the research (ChiCTR1900022959). We obtained written informed consent from all patients before the assessment. All stroke patients were diagnosed according to Chinese guidelines for the diagnosis and treatment of acute ischemic stroke in 2018. The inclusion criteria for ischemic stroke patients are detailed as follows: (1) age more than 18 years old; (2) no serious aphasia, speech expression, or communication barriers, can communicate with the researcher through words or body language; (3) time of stroke onset is known more than 2 weeks; (4) the modified Ranking score less than 3 and Barthel index score more than 20; (5) informed consent and volunteer to participate in the study. Patients were excluded if they met any of the following conditions: (1) with moderate or severe encephalatrophy; (2) with cognitive impairment or mental illness previously; (3) with malignant tumor, hematonosis, serious heart, liver, lung, and kidney disease; (4) dwelling in rehabilitation institutions; and (5) quitting during the investigation process.

### 2.2. Variables and Measurements

#### 2.2.1. Demographic Data

The general demographic data of patients includes gender, age, educational level, marital status, working status, body mass index (BMI), payment way for medical expenses, average monthly income of the family, and so on.

#### 2.2.2. Disease Related Data

Disease-related data, including smoking, drinking, chronic disease, family history of stroke, recurrence frequency, and health education about self-management before discharge.

#### 2.2.3. Self-Efficacy Data

The stroke self-efficacy questionnaire (SSEQ) was compiled by Jones and translated into Chinese by Li Hongyan in 2015 with good reliability and validity [8]. The revised Chinese version of the SSEQ includes 2 dimensions and 11 items, including items on activity function efficacy (6 items) and self-management efficacy (5 items). The total Cronbach's *α* coefficient of the scale is 0.969 [9]. Each item is scored by 0–10 grades. The higher score in each dimension indicates better self-efficacy, respectively. In order to classify the efficacy, we used the standard score index to make it comparable: the standard score index = (the actual score of this dimension/the highest score of the dimension) *∗* 100%; the score index less than 60% indicates poor efficacy, 60 to 80% indicates medium efficacy, and more than 80% indicates good efficacy.

#### 2.2.4. Disease Cognition Data

With the in-depth study of disease cognition, Broadbent evolved from the revised cognition questionnaire compiled by Weinman et al. [10] and Rona et al. [11], and the brief cognition questionnaire (BIPQ) was completed in 2006. The questionnaire includes 8 dimensions (items 1 to 8): consequences, timeline, personal control, treatment control, identity, concerns, understanding, and emotional response. Item 9 of the BIPQ is an open question that explores causal representation and assesses the top three causes of stroke. The higher score means the more negative disease cognition, which can quickly and conveniently evaluate the patients' status of disease cognition [12, 13].

### 2.3. Disability Level and the Ability of Daily Living

#### 2.3.1. Modified Rankin Scale (mRS)

Warlow designed mRS based on Rankin scale in 1988 to evaluate the disability level after stroke comprehensively. In this study, mRS was used to measure the ability to live independently and was divided into 7 levels: 0 for asymptomatic (no need for assistance), 5 for severe disability (complete dependence), and 6 for death. The scale has been widely used and has good reliability and validity [14].

#### 2.3.2. The Barthel Index (BI)

The Barthel index (BI) was published in 1965 by American scholars Florence Mahoney and Dorothea Barthel. It includes 10 items with a full score of 100. The lower the score is, the more serious the disability is, and the more help is needed. 0–20 points reflect extremely serious functional defects; 25–45 points reflect serious dysfunction; 50–70 points reflect moderate dysfunction; 75–95 points reflect mild dysfunction; and 100 points reflect complete self-care [15].

#### 2.3.3. Self-Management Data

The stroke self-management scale (SSMS) was designed by Wang Yanqiao, a Chinese scholar, on the basis of the self-management connotation and other chronic disease self-management behavior scales. It has seven dimensions, including disease management, medication management, diet management, daily life management, emotion management, social function and interpersonal management, rehabilitation, and exercise management. Out of the total of 50 items, 49 items were scored using a 5-level scoring method (1–5 points). In the dimension of food management, 1 item scored 1–10 points. The higher total score shows better self-management behavior. The total Cronbach's *α* coefficient of the scale is 0.835, the content validity is 0.95, and the structural validity is 0.594–0.771 [16]. In order to compare the scores of different dimensions, the standard score index is used. The standard score index = (the actual score of this dimension/the highest score of the dimension) *∗* 100%, the standard score index less than 60% indicates poor self-management, 60 to 80% indicates the medium self-management, and more than 80% indicates the good self-management.

### 2.4. Statistical Analysis

#### 2.4.1. Study Sample Size

PASS 11.0 software (NCSS., Rijswijk, The Netherlands) was used to calculate the sample size. 318 cases that need to be investigated. Assuming 10% nonresponse rate, the final sample size became 354.

#### 2.4.2. Data Analysis

Data input was performed using EpiData 3.1 (The Epidata Association, Odense, Denmark), and statistical analysis was performed using SPSS 19.0 (IBM Corp., Armonk, NY).

The classification data were described by frequency and proportion. The continuous data were described by means and standard deviation if they conform to normal distribution. If they do not conformed to a normal distribution, they are described by means of median and interquartile range.

Classification data were conducted by chi-square test or Fisher exact test. Continuous data were conducted by independent sample *t*-test or Mann–Whitney *U*-test. The variables of *P*  <  0.15 in single factor analysis and those closely related to self-management in clinical settings served as the independent variables for binary logistic regression analysis. Box Tidwell was used to test whether the logit conversion value between a continuous independent variable and a dependent variable is linear.

## 3. Results and Discussion

### 3.1. Data Collection

**Figure 1:**
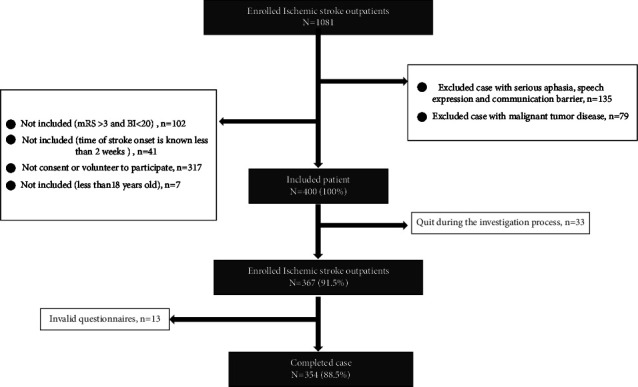
Research flow chart.

In this study, 400 questionnaires were sent out, 367 questionnaires were taken back, the response rate was 91.75%, 13 invalid questionnaires were eliminated, the remaining 354 valid questionnaires were left, and the effective rate was 96.45%. Please see Figure 1 research flow chart for details.

### 3.2. Demographic Characteristics

As shown in Table 1, this study included 354 effective stroke patients, of whom 108 (30.5%) were women and 246 (69.5%) were men, with an average age of 61.16 ± 13.01 years.

### 3.3. Diseases-Related Characteristics

Of the 354 patients who were included, 192 (54.2%) patients never smoke, 107 (30.2%) patients have quit smoking, and 206 (58.2%) patients never drink alcohol. The most common chronic disease was hypertension (58.8%). In this study, 50.8% of patients had no primary caregiver. The most common sequelae were motor dysfunction (27.1%). 46.9% of patients did not receive self-management health education before discharge. See Table 2 for details.

The disability level and activities of daily living are shown in Table 3. Of the 354 patients who were included, 75.7% patients in our study could completely self-care.

### 3.4. Descriptive Analysis of Self-Management Behavior of Stroke Patients

According to the Shapiro–Wilk normal test, the data conformed to the normal distribution. The score of the SSMS was 151.07 ± 18.53; 210 patients (59.3%) had poor self-management behavior, 141 patients (39.8%) were at medium level, and 3 patients (0.8%) had good self-management behavior. See Table 4 for details.

### 3.5. Single Factor Analysis

Since only three patients in the good level group, so we combined the good and middle group. Comparing the difference of self-management behavior between middle-high-level group and low-level group.

Tables 5 and 6 showed that the educational level, average monthly income of the family, payment method of medical expenses, health education of self-management before discharge, SSEQ self-management efficacy dimension, and the score of SSEQ and BIPQ between the two groups were statistically significant (*P*  <  0.05).

### 3.6. Multivariable Analysis

21 items were included in the linear test model, and the significance level after Bonferroni correction was 0.0024. The linear test results showed that there is a linear relationship between all the continuous independent variables and the logit conversion value of the dependent variable.

Finally, the logistic model was statistically significant (*χ*^2^ = 53.876, *P*  <  0.001) (Table 7). The model can correctly classify 66.9% of the research objects. Among the variables included in the model were statistical significance (*P*  <  0.05) in terms of payment method for medical expenses, SSEQ self-management efficacy, self-management health education before discharge, age (old age), educational level, and mRS score. See Table 7 for details.

## 4. Discussion

This study reported the actual whole status of self-management behavior and studied the risk factors of self-management in Chinese stroke patients. The results indicated that the payment way of medical expense, self-management efficiency, health education about self-management before discharge, age, educational level, and mRS score were independently influencing factors of self-management behavior.

Although Healthy China 2030 emphasized the importance of structuring a better health security system, especially for chronic diseases [17], patients still need to pay a part of their medical bills, even if they have medical insurance. Our study shows socioeconomic factors such as inadequate health insurance may have a negative impact on self-management behavior. Our study is consistent with the national registry study in China, which demonstrated that health insurance status was significantly associated with 1-year outcomes for patients with stroke. Patients who are covered by the New Rural Cooperative Medical Scheme, which has lower reimbursement rates than the Urban Basic Medical Insurance Scheme [18]. However, stroke is a leading cause of long-term disability globally and a resource-intensive disease, both directly and indirectly [19]. A Sweden study based on regional administrative systems found that the monetary burden of stroke is very high, and patients who are affiliated with stroke may also have a socioeconomic gradient in the utilization of Swedish health insurance [20]. Prior studies have demonstrated that insurance status is an independent predictor of patient safety events after stroke. The absence of private insurance is associated with higher mortality, longer lengths of stay, and worse clinical outcomes [21]. Thus, it is important to call for more attention and more resources for stroke. It is an urgent task around the world to increase economic investment, expand the coverage of medical insurance, and increase the proportion of reimbursement for major diseases such as stroke.

Patients with high efficiency in self-management are more likely to have better self-management behaviors. More and more studies have pointed out the importance of self-efficacy in the long-term care of people with enduring illnesses; patients with high efficacy function better in daily activities, perceive a better quality of life, and have worse emotional disorders than patients with low self-efficacy [22–24]. Among many theories on behavior change, Social Cognitive Theory emphasizes it is a multifaceted causal structure in which self-efficacy plays an important role in this process[25]. Actually suffering from stroke may be a traumatic experience for patients, it is a huge challenge for them. A study shows that perceived coping self-efficacy emerges as a focal mediator of posttraumatic recovery, which lends support to the centrality of the enabling and protective function of belief in one's capability to exercise some measure of control over traumatic adversity [26]. Nowadays, some interventions based on self-efficacy aiming at improving outcomes in stroke patients are showing up gradually [27–29]. Thus, we should explore more effective interventions to improve self-management efficiency, especially for those needing long-term care diseases, eliminate the negative emotions and pessimistic attitude, so as to increase the possibility to change unhealthy lifestyle and improve self-management ability.

Patients who accepted the self-management health education before discharge may have better self-management behaviors. Health education is a continuous, dynamic, complex, and planned teaching process throughout the whole process of life [30]. Health education could help patients and their caregivers know the right knowledge about this disease and enhance their faith in changing bad behaviors. A study found that nurses can help improve patients' knowledge and cognition of the risks of stroke by playing the health education CD-ROM and providing printed information during the patients' wait time before appointments [31]. In our study, this question was reported by themselves, nearly half of the patients answered they didn't receive the content of stroke in hospital. On the one hand, patients may have a misperception due to low sensitivity and acceptance of health knowledge. On the other hand, the information we provided to patients they may don't need. However, providing health education about how to manage themselves before discharge is the theoretical basis for long-termin-home management. With the development of the times, more and more health education models emerged. The health belief model is generally used to reduce the risk of developing a disease or manage an already existing disease. According to the health belief model, patients may change their behavior if they perceive a health threat [32]. Besides, social cognitive theory also posits that health behaviour is determined by the interaction of personal cognitive factors, socioenvironmental factors, and behavioral factors [33]. Therefore, we should formulate the content and form of health education under the guidance of these health education theories, taking into account the patients' cognitive, social, and economic factors, individual factors, and other specific factors. The form of health education is also changing with time; social media has now become a popular and broad way to do so as the world continues to advance technologically [34]. It is a global trend to transform traditional health education into digital education. So, future research can also continue to explore the form and effect of health education with the help of artificial intelligence and other new technologies.

Age is an unalterable factor for self-management behavior; our study found that the elderly (age more than 60 years) may have better self-management behavior compared to the young (less than 44 years). The elderly patients, especially the retired, have more time and energy to manage their own diseases. While the young who have a long life expectancy after stroke may consider return to the society more. It is hard for the young population to devote themselves to long-term rehabilitation as peers all working and taking family responsibilities. With an increasing incidence of stroke in young adults, the optimal and specific management of this population needs more research studies.

In our study, patients with better limb function had better self-management behaviors than those who had a severer motor disability. Physical well-being seems to be the most affected component of quality of life. A review showed that the level of disability, presence of comorbidities, and motor function were the key predictors for quality of life among stroke survivors in Africa [35]. The lives of patients with better limb function are less affected, which in turn will promote these patients to better implement self-management behavior. Naturally, patients with better limb function have fewer difficulties with self-management, so they can change their poor lifestyle and improve their self-management abilities faster. Nowadays, more and more studies are focusing on different interventions to improve the motor function of stroke patients, including repetitive transcranial magnetic stimulation [36], virtual reality [37], mirror therapy [38], and so on. Within these fields, upper limb function is the most concerning.

In general, the self-management behavior among Chinese stroke patients is at the middle level. The independent factors that affect the self-management behavior of stroke patients are: the payment way for medical expenses, SSEQ self-management efficacy, self-management health education before discharge, age (elderly), educational level, and mRS. It is not only necessary to popularize the knowledge of basic prevention and control of the disease, but it is also necessary to continuously encourage the patients to establish the determination and confidence to effectively control the disease and gradually improve self-efficacy, so as to change the unhealthy behaviors affecting health and finally improve self-management ability.

## 5. Limitations

This study has some limitations. First, because the survey was finished in one hospital, the representativeness of the samples was limited. Second, this study only investigated the score of mRS less than 3. Some patients with severe disabilities still have part of self-management ability, which needs further research to explore the self-management ability of patients with severe disabilities. Third, the regression models we used may have several limitations in determining the risk factor of the given variable. Further studies may use a muticenter, large survey to verify our conclusion. If possible, use the SEMs to discover the severity and direction of the association between self-management and other factors.

## 6. Conclusions

The self-management behavior of Chinese stroke patients is at the middle level. Patients with medical insurance, high self-management efficiency, and better limb function may have better self-management behavior. Besides, patients with a high educational level who accept health education before discharge may also have better self-management behavior. It is important for patients to get knowledge about stroke in a right way and set up the faith to take care of themselves independently gradually. Furthermore, it is necessary and important for medical staff to give all patients health education about self-management before discharge and to increase the effectiveness of interventions for rehabilitation.

## Figures and Tables

**Table 1:** Demographic characteristics of the 354 effective patients.

Characteristic		*N*	%
Sex	Female	108	30.5
	Male	246	69.5

Working status	Not working/retired	185	52.3
	Employed	169	47.7

Educational level	Junior high school and below	216	61.0
	High school and above	138	39.0

Address	Rural	114	32.2
	City	240	67.8

Marital status	In relationship	319	90.1
	Not in relationship	35	9.9

Average monthly income of family (RMB)	≤3000	131	37.0
	3001∼6000	144	40.7
	>6000	79	22.3

BMI (kg/m^2^)	Low weight	9	2.5
	Normal weight	184	52.0
	Overweight	133	37.6
	Obesity	28	7.9

Payment method of medical expenses	Medical insurance	320	90.4
	At one's own	34	9.6

Primary caregiver	Husband	30	8.5
	Wife	103	29.1
	Offspring	28	7.9
	Grandchildren	4	1.1
	None	180	50.8
	Other	9	2.5

*Note.* BMI: body mass index.low weight (BMI < 18.5 kg/m^2^), normal weight (18.5 kg/m^2^ ≤ BMI < 24.0 kg/m^2^), and overweight/obesity (BMI ≥ 24.0 kg/m^2^).

**Table 2:** Clinical characteristics of the 354 effective patients.

Item		*N*	%
Smoking	Yes	55	15.5
	Never	192	54.2
	Quit	107	30.3

Drinking alcohol	Everyday	24	6.7
	Every week	18	5.1
	Rarely	58	16.4
	Never	206	58.2
	Quit	48	13.6

Hypertension	No	146	41.2
	Yes	208	58.8

Diabetes	No	263	74.3
	Yes	91	25.7

Hyperlipidemia	No	215	60.7
	Yes	139	39.3

Obstructive sleep apnea syndrome	No	336	94.9
	Yes	18	5.1

Carotid atherosclerosis	No	279	78.8
	Yes	75	21.2

Atrial fibrillation	No	332	93.8
	Yes	22	6.2

Family history	No	283	79.9
	Yes	71	20.1

Duration, year	<1	195	55.1
	≥1	159	44.9

Recurrence rate	0	298	84.2
	1	41	11.6
	2	6	1.7
	≥3	9	2.5

Sequela	No	205	57.9
	Yes	149	42.1

Dysphagia	No	342	96.6
	Yes	12	3.4

Speech disorders	No	298	84.2
	Yes	56	15.8

Sensory dysfunction	No	333	94.1
	Yes	21	5.9

Motor dysfunction	No	258	72.9
	Yes	96	27.1

Cognitive impairment	No	341	96.3
	Yes	13	3.7

Self-management health education before discharge	No	166	46.9
	Yes	188	53.1

**Table 3:** The disability level and activities of daily living level of the 354 effective patients.

Item		*N*	%
mRS	0	128	36.1
	1	174	49.2
	2	36	10.2
	3	16	4.5

BI	Severe dysfunction	1	0.3
	Moderate dysfunction	4	1.1
	Mild dysfunction	81	22.9
	Fully self-care	268	75.7

*Note.* mRS: modified Rankin scale, BI : the Barthel index.

**Table 4:** The characteristics of self-management behavior of the 354 effective patients.

Dimension	Total score	Mean	Standard deviation	Standard score index (%)
Disease management	8442	23.85	8.70	43.36
Drug administration	5270	14.89	2.66	59.55
Diet management	9157	25.87	3.82	57.48
Daily living management	8988	25.39	4.70	63.47
Emotion management	5955	16.82	2.38	67.29
Social function and interpersonal management	7829	22.12	4.69	73.72
Rehabilitation exercise management	7837	22.14	6.33	63.25
Total score	53478	151.07	18.53	59.24

**Table 5:** Characteristics of poor group versus middle-high group (classification data).

Item		Poor	Middle-high	*P* value
Sex	Female	62	46	0.627^a^
	Male	148	98	

Working status	Not working/retired	109	76	0.872^a^
	Employed	101	68	

Educational level	Junior high school and below	144	72	**≤0.001^a^**
	High school and above	66	72	

Address	Rural	76	38	0.053^a^
	City	134	106	

Marital status	Not in relationship	24	11	0.241^a^
	In relationship	186	133	

Average monthly income of family (RMB)	≤3000	85	46	**0.016^a^**
	3001∼6000	89	55	
	>6000	36	43	

BMI (kg/m^2^)	Low weight	5	4	0.504^b^
	Normal weight	114	68	
	Overweight	75	57	
	Obesity	14	14	

Payment method of medical expenses	Medical insurance	184	136	**0.032^a^**
	At one's own	26	8	

Smoking	Yes	36	19	0.348^a^
	Never	116	76	
	Quit	58	49	

Drinking alcohol	Every day	13	11	0.523^a^
	Every week	12	6	
	Rarely	33	25	
	Never	128	78	
	Quit	24	24	

Hypertension	No	92	54	0.236^a^
	Yes	118	90	

Diabetes	No	152	111	0.320^a^
	Yes	58	33	

Hyperlipidemia	No	126	89	0.733^a^
	Yes	84	55	

Obstructive sleep apnea syndrome	No	197	139	0.253^a^
	Yes	13	5	

Carotid atherosclerosis	No	170	109	0.234^a^
	Yes	40	35	

Atrial fibrillation	No	198	134	0.638^a^
	Yes	12	10	

Primary caregiver	No	111	69	0.361^a^
	Yes	99	75	

Duration, year	<1	109	86	0.146^a^
	≥1	101	58	

Recurrence rate	0	181	117	0.211^a^
	≥1	29	27	

Sequela	No	116	89	0.219^a^
	Yes	94	55	

Dysphagia	No	203	139	1.000^b^
	Yes	7	5	

Speech disorders	No	173	125	0.262^a^
	Yes	37	19	

Sensory dysfunction	No	200	133	0.260^a^
	Yes	10	11	

Motor dysfunction	No	145	113	**0.050^a^**
	Yes	65	31	

Cognitive impairment	No	204	137	0.325^a^
	Yes	6	7	

Self-management health education before discharge	No	114	52	**≤0.001^a^**
	Yes	96	92	

mRS	0	67	61	0.080^a^
	1	105	69	
	2	26	10	
	3	12	4	

BI	Severe dysfunction	1	0	0.429^b^
	Moderate dysfunction	3	1	
	Mild dysfunction	53	28	
	Fully self-care	153	115	

SSEQ activities daily living effectiveness	Low self-efficacy	12	5	**0.029^a^**
	Medium self-efficacy	67	30	
	High self-efficacy	131	109	

SSEQ self-management effectiveness	Low self-efficacy	61	21	**≤0.001^a^**
	Medium self-efficacy	80	45	
	High self-efficacy	69	78	

*Note*. BMI: body mass index, low weight (BMI < 18.5 kg/m^2^), normal weight (18.5 kg/m^2^ ≤ BMI < 24.0 kg/m^2^), and overweight/obesity (BMI ≥24.0 kg/m^2^). mRS: modified Rankin scale, BI: the Barthel index, SSEQ: the stroke self-efficacy questionnaire. a: Pearson Chi-square, b: Mann–Whitney-*U* test. Bold stands for *p* value <0.05.

**Table 6:** Characteristics of poor group versus middle-high group (continuous data).

Item	Poor	Middle-high	*P*
Age, year (mean ± SD)	60.30 ± 13.42	62.41 ± 12.34	0.133^a^
BMI, kg/m^2^ (mean ± SD)	23.69 ± 2.78	23.97 ± 2.97	0.373^a^
BIPQ (mean ± SD)	39.96 ± 9.64	36.67 ± 9.61	**0.002^a^**

*Note.* BMI: body mass index, BIPQ: the brief cognition questionnaire. a: Pearson's chi-square, bold stands for *P* value <0.05.

**Table 7:** The multifactor analysis of stroke self-management behavior in 354 patients.

Item	B	S.E	Wals	*P*	OR	95 CI	95 CI
Payment way of medical expenses	1.168	0.450	6.728	0.009	3.215	1.330	7.769
SSEQ self-management effectiveness	0.903	0.242	13.883	<0.001	2.467	1.534	3.968
Self-management health education before discharge	0.856	0.245	12.234	<0.001	2.354	1.457	3.802
Age	0.723	0.249	8.435	0.004	2.060	1.265	3.355
Educational level	0.626	0.239	6.833	0.009	1.869	1.169	2.988
mRS score 0	0.615	0.252	5.957	0.015	1.850	1.129	3.031
Constant	3.223	0.556	33.652	<0.001	0.040		

*Note.* SSEQ: the stroke self-efficacy questionnaire, mRS: modified Rankin scale, BI: the Barthel index.

## Data Availability

The data that support the findings of this study are available from the corresponding author.
